# Chemical-induced heart defects using a transgenic zebrafish model

**DOI:** 10.1093/toxsci/kfaf083

**Published:** 2025-06-12

**Authors:** Shujie Liu, Toru Kawanishi, Atsuko Shimada, Yuko Nukada, Masaaki Miyazawa, Hiroyuki Takeda, Junichi Tasaki

**Affiliations:** R&D, Safety Science Research, Kao Corporation, Kanagawa 210-0821, Japan; Department of Biological Sciences, Graduate School of Science, University of Tokyo, Tokyo 113-0033, Japan; Department of Biological Sciences, Graduate School of Science, University of Tokyo, Tokyo 113-0033, Japan; R&D, Safety Science Research, Kao Corporation, Tochigi 321-3497, Japan; R&D, Safety Science Research, Kao Corporation, Tochigi 321-3497, Japan; Department of Live Sciences, Kyoto Sangyo University, Kyoto 603-8555, Japan; R&D, Safety Science Research, Kao Corporation, Kanagawa 210-0821, Japan

**Keywords:** heart defects, teratogen, cardiomyocyte, endocardium, cardiotoxicity, transgenic zebrafish model

## Abstract

Congenital heart defects (CHDs) are common birth defects attributed to genetic and environmental factors, such as pharmaceuticals and chemicals. Identifying modifiable environmental factors and understanding their impact on heart development is crucial for mitigating chemical-induced CHDs. Given the increasing number of chemical agents, efficient high-throughput systems are essential to evaluate their teratogenic potential during cardiovascular development, which is a major concern for chemical safety. In this study, we developed 3 transgenic zebrafish reporter lines, *myl7:EGFP*, *kdrl:mRFP*, and *gata1:mKate2*, which enable real-time visualization of myocardial and endocardial development and cardiac function based on blood flow. These transgenic embryos were used to investigate the teratogenic effects of chemicals well known to induce heart defects in mammals, including humans. Our real-time imaging revealed that the teratogens induced significant malformations in cardiac morphogenesis, including abnormal heart tube formation, incomplete cardiac looping, and reduced heart chamber size. These teratogens also disrupted the expression of cardiac progenitor markers, suggesting impaired cardiac progenitor development. These defects were detected at the early stages (4–48 h post-fertilization), suggesting that the stages of progenitor development to heart looping were most susceptible to teratogen exposure, i.e. the critical period for teratogen-induced heart defects. Functional defects, such as impaired blood flow, were observed using real-time imaging of the *gata1*-reporter line. This study demonstrates the utilization of transgenic zebrafish embryo models for high-throughput teratogenicity testing, which also allows us to analyze the mechanisms underlying chemical-induced heart defects. Therefore, our zebrafish models would contribute to the identification and reduction of risks associated with CHDs.

Congenital heart defects (CHDs) are one of the most frequent congenital birth defects worldwide (Liu et al. 2019). These defects are defined as structural malformations of the heart and/or blood vessels. In addition to genetic factors, environmental factors such as pharmaceuticals and chemicals are known to cause heart defects. Subtle perturbations caused by environmental factors could result in significant CHDs and potentially lead to embryonic or fetal death (Lynch and Abel 2015; Kalisch-Smith et al. 2020). These environmental factors are modifiable and can be mitigated (Wilson et al. 1998; Jenkins et al. 2007) and will therefore be the target of studies to reduce the incidence of heart defects. Indeed, significant progress has been made over the past decade in identifying the causative genes for CHDs (Fahed et al. 2013; Pierpont et al. 2018), and information about environmental factors also has accumulated, which can adversely affect the developing heart. However, we still have limited information on how to manage environmental risk factors based on experimental evidence (Jenkins et al. 2007; Mahler and Butcher 2011). Thus, to reduce the risk of chemical-induced CHDs, it is crucial to establish a reliable assay system with which to analyze their mechanisms.

Teratogenicity, the potential of teratogens to induce developmental malformations, has been evaluated in the context of developmental toxicity tests. Although rodents and rabbits, among other mammals, have been utilized for these tests, the current priority on the 3Rs (replacement, reduction, and refinement) and the conservation of resources has led to the development of alternative approaches for assessing teratogenicity testing. Therefore, a teratogenic evaluation system for heart defects must meet the criteria of efficiency (i.e. high-throughput) and the ability to provide mechanistic insights, based on the 3R principle.

Various model organisms, including zebrafish, frogs, and chicks, have been extensively utilized in cardiovascular developmental biology as models of mammalian systems (Warkman and Krieg 2007; Bakkers 2011; Kaltenbrun et al. 2011; Wittig and Münsterberg 2020; Bowley et al. 2022). Despite the structural differences between two-chamber and four-chamber hearts, cardiac development remains highly conserved among vertebrates (Lohr and Yost 2000; Stainier 2001; Bakkers 2011). Thus, a comprehensive understanding of normal cardiac development is essential to elucidate the mechanisms underlying teratogen-induced heart defects and to translate findings from models to human CHD research.

Recently, zebrafish have gained significant attention as a model organism in toxicology and as a promising alternative for teratogenicity testing. Consequently, various zebrafish-based teratogenicity assays have been developed, including a standard teratogenicity assay (Brannen et al. 2010), an automated assay (Teixidó et al. 2019), and validation studies for these assays (Song et al. 2021; Weiner et al. 2024). Zebrafish have several advantages for high-throughput genetic and chemical screening, including evolutionarily conserved developmental programs, various genetic manipulation tools, and rapid external development with high transparency. In particular, a large number of mutants affecting heart development have been reported, which are excellent resources to understand the mechanisms of normal cardiac development (Chen et al. 1996; Stainier et al. 1996). Thus, they could also be complementary to chemical-induced heart defects in toxicology.

Evidence supports the high predictive ability of zebrafish models for teratogenicity evaluation (Brannen et al. 2010; Selderslaghs et al. 2012; Augustine-Rauch et al. 2016; Inoue et al. 2016). However, the systems using conventional wild-type zebrafish have difficulty in detecting defects in the heart and blood vessels, paradoxically, due to their transparency. This limits the availability of the zebrafish systems in terms of time and accuracy of output testing. Recently, we developed a teratogenicity assay system using transgenic zebrafish lines to improve the detection and analysis of chemical-induced craniofacial anomalies (Liu et al. 2023), in which craniofacial skeletons are specifically labeled with EGFP. Compared with the conventional system, these lines increase the efficiency and accuracy of craniofacial malformation detection, along with the understanding of the underlying mechanisms. Furthermore, given the high visibility of labeled organs in this zebrafish system, we were able to determine the time window for adverse effects of teratogens on craniofacial development (i.e. the critical period), which will lead to the establishment of risk management protocols.

Here, we adopted the above similar strategy to investigate heart defects and developed a series of transgenic zebrafish lines to visualize heart development and function. Indeed, previous studies using transgenic reporter lines primarily focused on cardiotoxicity and/or the environmental impact of chemicals (Chen et al. 2019; Dyballa et al. 2019; Lei et al. 2024). In contrast, our study focuses on the developmental processes leading to abnormal heart morphology, utilizing live-imaging data across 7 developmental stages, from the progenitor stage to valvular formation. We first described embryonic heart development using these lines and treated their embryos with teratogens known to induce heart defects in mammals to examine their teratogenic effects on heart development and function. We also analyzed the critical period for heart defects in zebrafish embryos. This study proposes that the series of transgenic lines generated herein is an ideal platform for evaluating teratogenicity in fish heart development, the results of which could be extrapolated to the mammalian heart.

## Materials and methods

### Zebrafish transgenesis and maintenance

The Tol2 transposon system was used for the transgenesis (Suster et al. 2009). Transgenesis was performed as previously described (Liu et al. 2023). The promoter region of *myl7*, *kdrl*, and *gata1* was isolated from the RIKEN WT (RW) strain. The length of the promoter region was as follows: *myl7* (1.1 kb), *kdrl* (6.3 and 6.8 kb), and *gata1* (8.1 kb). Each transposon construct was injected with Tol2 transposase mRNA at the one-cell embryo stage. The zebrafish (*Danio rerio*) strain RW, *Tg(−1*.*1myl7:EGFP)*, *Tg(−6*.*3kdrl:mRFP)*, *Tg(−6*.*8kdrl:mRFP)*, and *Tg(−8*.*0gata1:mKate2)* (RW background) were maintained with a 14-h light/10-h dark cycle. The water temperature was kept at 28 ± 1 °C, and quality conditions were maintained according to the Zebrafish Book (Westerfield 2000) and the Guide for the Care and Use of Laboratory Animals 8th edition (National Research Council 2011).

### Egg production and embryo exposure

Adult male and female zebrafish were placed in a breeding tank with a separator in the late afternoon before spawning. The separator was removed in the morning, and spawning was stimulated when the light was turned on. The fertilized eggs were collected within 1 h after removing the separator. The eggs were incubated in E3 medium (5 mM NaCl, 0.17 mM KCl, 0.33 mM CaCl_2_, 0.33 mM MgSO_4_, and 0.1 mM NaOH) at 28 °C before chemical exposure. Thirty fertilized eggs were placed in a six-well plate with 3 ml of exposure medium. Chemical treatments were administered from 4 to 96 h post-fertilization (hpf), with the exposure medium replaced daily. The test compounds were dissolved in either distilled water (valproic acid [VPA]) or dimethyl sulfoxide (DMSO; aspirin [ASP], warfarin [WA], retinoic acid [RA], and methotrexate [MTX]), with the final DMSO concentration in the exposure medium maintained at 0.1%. Samples were collected from the 13 somite stage (ss) until 96 hpf.

### Test compounds

Before conducting the exposure experiments, a dose range-finding study was performed using 4 different concentrations of each chemical: 1 μM, 10 μM, 100 μM, and 1 mM. The final exposure concentration was selected based on 2 criteria: (i) 100% embryo viability and (ii) the concentration that induced the most severe and frequent phenotype. This selection approach follows the methodology established in our previous studies (Liu et al. 2020; Narumi et al. 2020; Liu et al. 2023).

After the dose range-finding study, exposure dose was determined as follows: ASP (30, 60, 80 µM, Wako), WA (10, 20, 40 µM, Wako), RA (2.5, 5, 10 nM, Tokyo Chemical Industry), and MTX (50, 100, 200 µM, Wako), which were diluted from stock solutions prepared with DMSO (Wako). VPA (7.5, 15, 30 µM, Wako) was diluted from stock solutions prepared with distilled water (Life Technologies). The concentrations shown in the figures are non-lethal and represent the doses at which the most phenotypes were observed: ASP (60 µM), VPA (15 µM), WA (20 µM), RA (5 nM), and MTX (200 µM) (Table 1).

**Table 1.** Compounds used in the study.

Category	Test compound	Abbreviation	CAS No.	Supplier	Solvent	Concentration (µM)
Nonsteroidal anti-inflammatory drugs	Aspirin	ASP	50-78-2	Wako	DMSO	60^a^
Antiepileptic agents, anticonvulsants	Valproic acid	VPA	99-66-1	Wako	Distilled water	15
Cardiovascular agents, antithrombotic agents, anticoagulants	Warfarin	WA	129-06-6	Wako	DMSO	20
Vitamin A, topical use in acne	Retinoic acid	RA	302-79-4	Tokyo Chemical Industry	DMSO	5×10^–2^
Antineoplastic agents, immunomodulating agents	Methotrexate	MTX	302-79-4	Wako	DMSO	200

a Highest viable concentration.

### Whole-mount in situ hybridization

Whole-mount in situ hybridization was performed as previously described (Liu et al. 2025). Zebrafish RNA probes of *nkx2.5* and *islet1a* (*isl1a)* for in situ hybridization were synthesized according to the following protocols: Partial cDNA sequences of 1,000–1,500 bases with T7 and T3 promoter sequences as templates for RNA probes were synthesized using integrated DNA technology. For template synthesis of each probe by PCR, the following primers were used: T7 promoter primer (5′-CCCTATAGTGAGTCGTATTA-3′) and T3 promoter primer (5′-ATTAACCCTCACTAAAGGGAA-3′). Antisense riboprobes were synthesized using T7 MEGASCRIPT using DIG-labeled UTP (Roche), according to the manufacturer’s protocol.

Zebrafish embryos were fixed at 13 ss for 2 h with 4% paraformaldehyde (PFA) (Wako) and dehydrated for >2 h in ice-cold methanol (MeOH, Wako) at −20 °C. Embryos were rehydrated stepwise with 75%, 50%, and 25% MeOH in PBS-T, phosphate-buffered saline (PBS, Invitrogen) containing 0.1% Triton X-100 (Cayman Chemical) on ice, and placed in PBS-T. The samples were incubated with 10 µg/ml of protease type XIV (Sigma-Aldrich) in PBS-T for 10 min and post-fixed in 4% PFA for 20 min. After post-fixation, the samples were washed twice with PBS-T for 5 min. The embryos were prehybridized for at least 1 h at 60 °C in a hybridization buffer (50% formamide [Nacalai Tesque], 10% dextran sulfate [Sigma-Aldrich], 5× saline-sodium citrate [SSC] pH 7.0 [Nippon Gene], 10% sodium dodecyl sulfate [SDS, Wako], 50 mg/ml heparin [Sigma-Aldrich], 50 mg/ml tRNA [Roche], and 0.1% Tween-20). Hybridization was performed in the hybridization buffer containing 500 ng of the RNA probe overnight at 60 °C. Samples were washed twice with wash buffer I (50% hybridization buffer, 2× SSC [pH 4.5], 1% SDS, and 0.1% Tween 20) for 15 min at 60 °C and twice with wash buffer II containing 500 mM NaCl, 10 mM Tris-HCl, and 0.1% Tween 20 for 15 min at 60 °C. The samples were subsequently blocked with 2% goat serum (Gibco) and 2 mg/ml bovine serum albumin (BSA, Wako) in PBS-T for 2 h and incubated overnight at 4 °C with the preabsorbed alkaline phosphatase-coupled anti-digoxigenin antiserum (Roche) at a 1:5,000 dilution in the blocking buffer. Finally, the samples were washed 6 times with PBS-T for 15 min. The detection solution (450 mg/ml NBT and 175 mg/ml BCIP, Roche) in the alkaline phosphatase reaction buffer (100 mM Tris-HCl [pH 9.5], 50 mM MgCl_2_, 100 mM NaCl, and 0.1% Tween 20) was used. The samples were washed thrice with PBS-T for 5 min, following which the reaction was terminated by incubating with 4% PFA for 30 min. All samples were transferred to 90% glycerol in PBS for observation and imaged using Leica M80 or Stemi305.

### Immunofluorescent staining and fluorescence imaging

Immunofluorescent staining was performed as previously described in Narumi et al. (2020). Zebrafish embryos were fixed with 4% PFA at 96 hpf, treated with 100% ice-cold MeOH (Wako) to achieve dehydration, and placed at −20 °C for longer storage. The samples were processed to remove the pigmentation by bleaching with 3% hydrogen peroxide (Wako) and 0.5% potassium hydroxide (Wako) under light for 2 h. After bleaching, the samples were incubated with 10 µg/ml of protease type XIV in PBS-T for 30 min. The samples were post-fixed in 4% PFA for 20 min. After post-fixation, samples were washed with 150 mM Tris–HCl (pH 8.5) for 5 min, then heated for 15 min at 70 °C, and washed twice with PBS-T for 5 min. The samples were then incubated in ice-cold acetone (Wako) for 20 min at −20 °C and blocked using 3% BSA in PBS-T for 2 h.

The samples were incubated with mouse anti-GFP (1:1,000, Invitrogen: AB_221568; or 1:1,000, EMD Millipore: MAB3580) and rabbit anti-tRFP (1:1,000, Everogen: AB233) overnight at 4 °C. The samples were washed 6 times with PBS-T for 15 min and stained with the following secondary antibodies: Alexa Fluor 488-goat anti-mouse, Alexa Fluor 568-goat anti-rabbit, secondary antibodies (1:1,000, Life Technologies), and DAPI solution (1:1,000, DOJINDO) overnight at 4 °C. After washing 6 times with PBS-T for 15 min, the samples were embedded in 1% low-melting agarose and mounted on a 35-mm non-coated glass-bottomed dish (IWAKI).

For live imaging, to inhibit pigmentation, zebrafish embryos were treated with 0.003% 1-phenyl-2-thiourea (PTU; Sigma) starting at 24 hpf. PTU was administered for 2 h on the mornings of days 1 to 3 post-fertilization, following the method described by Isogai et al. (2001).

Additionally, embryos were treated with 0.2% MS222 and mounted in 0.01% low-melting agarose containing 0.2% MS222 to temporarily stop heartbeats, following the protocol described by Felker et al. (2018). The samples were anesthetized with 0.02% MS-222 (Sigma-Aldrich) at 18 ss (18 hpf) and 0.2% MS-222 after 24 hpf before sample mounting. Confocal live imaging was performed as previously described (Hoshino et al. 2024). The samples were embedded in 1% low-melting agarose (Sigma-Aldrich) containing 0.02% or 0.2% MS-222 on a glass-bottomed dish. Live samples and fluorescent-stained samples were imaged on Zeiss LSM800 system equipped with Zeiss ZEN blue software and were presented as representative optical sections or maximum intensity projections. All procedures were performed at room temperature unless otherwise specified.

For live imaging of quantification, the samples were anesthetized with 0.02% MS-222 at 18 ss (18 hpf) and 0.2% MS-222 after 24 hpf before sample mounting. Thirty embryos were used for each teratogen treatment. However, embryos with proper positioning or orientation were used for quantification. For quantification of cardiac disc, heart tube, looping, and chamber formation, samples were mounted in 2% methyl cellulose (Sigma), and each structure was imaged using an MZ10 fluorescent stereomicroscope (Leica Microsystems). Images were subsequently analyzed using ImageJ (National Institutes of Health). To quantify the degree of cardiac looping, the angle formed between the ventricle and atrium was measured (see Fig. 5A'). The vertex of the angle was defined at the outer curvature in the ventricle. The looping angle was measured between 2 lines: One extending along the axis of the ventricle and the other toward the atrium (see Fig. 5A').

### Light sheet microscopy

*Tg(myl7:EGFP;gata1:mKate2)* embryos were anesthetized with 0.05% MS-222 until the fish stopped moving; however, the heart was maintained at a normal speed. They were then mounted in 0.8% low-melt agarose (Promega) in a glass capillary (inner diameter, 1 mm). The capillary was immersed in a water chamber at 28.5 °C and connected with the Lightsheet Z.1 microscope (Zeiss) equipped with a 1.0 NA/20× W Plan water dipping objective and a CCD camera. The sample was allowed to rotate so that the ventricle and atrium were in the focal plane. Images of the *z*-plane were continuously recorded for 3 or 4 cardiac cycles at a maximal frame rate of 5 frames per second. The laser excitation wavelengths were 488 and 561 nm for EGFP and mKate2, respectively.

### Statistics

For the quantification of each developmental event in cardiogenesis (i.e. cardiac cone formation, heart tube formation, looping, ballooning, and endocardial cushion formation) with dose–response analysis, multiple comparisons were conducted using GraphPad Prism version 8 for Windows (La Jolla, CA, USA), and *P*-values were calculated through one-way ANOVA followed by Dunnett’s multiple comparison test. Statistical significance was defined as *P* < 0.05. All data are expressed as the mean ± SD unless stated otherwise.

## Results

### Reporter lines to visualize the myocardium, endocardium, and endothelium

**Fig. 1.**
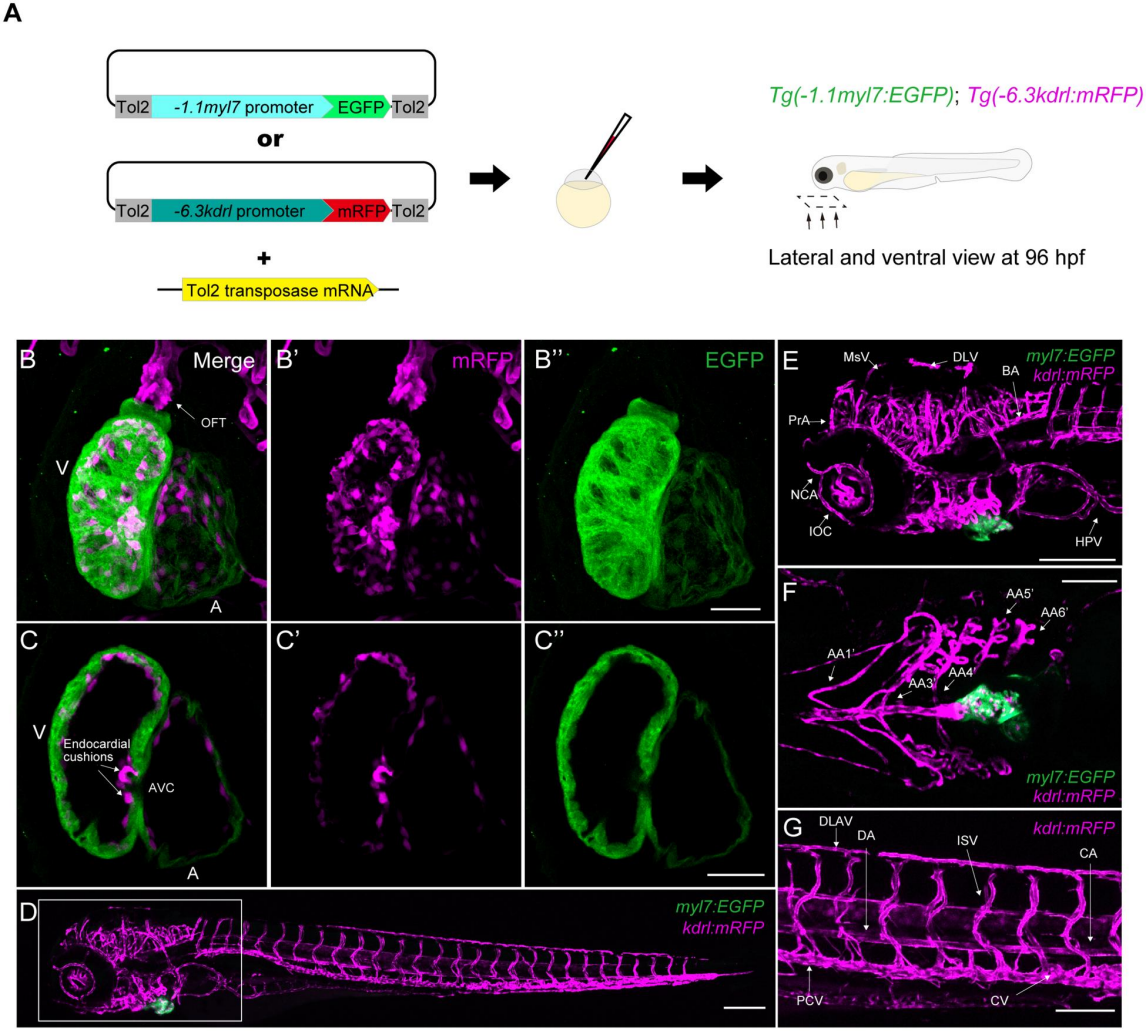
Generation and characterization of *myl7:EGFP*; *kdrl: mRFP* transgenic zebrafish. (A) Schematic diagram for generating *Tg(−1.1 myl7:EGFP)* and *Tg(−6.3kdrl:mRFP)*. (B to B") Immunofluorescence images of zebrafish heart at 96 hpf. Maximum intensity projection of z-stack images was performed. A: atrium; OFT: outflow tract; V: ventricle. (C to C") The optical section of the ventricle and atrium separated by the endocardial cushions at the atrioventricular canal (AVC). A: atrium; V: ventricle. (D) Live imaging of *myl7:EGFP; kdrl:mRFP* embryo at 96 hpf. (E) Magnified view of the rectangle in (D), BA: basilar artery; DLV: dorsal longitudinal vein; HPV: hepatic portal vein; IOC: inner optic circle; MsV: mesencephalic vein; NCA: nasal ciliary artery; PrA: prosencephalic artery. (F) Enlarged ventral view of the rectangle in (D), AA1': mandibular arch, AA3': first branchial arch, AA4': second branchial arch, AA5': third branchial arch, AA6': fourth branchial artery. (G) Magnified view in the tail region of *myl7:EGFP; kdrl:mRFP* at 96 hpf. CA: caudal artery; CV: caudal vein; DA: dorsal aorta; DLAV: dorsal longitudinal anastomotic vessel; PCV: posterior cardinal vein; ISV: intersegmental vessel. Scale bars: 50 μm in (B–C"); 100 μm in (D–G).

To visualize the anatomical structure of the heart in zebrafish embryos, we generated *Tg(−1.1myl7:EGFP)*, previously known as *cmlc2*, *Tg(−6.3kdrl:mRFP)*, and *Tg(−6.8kdrl:mRPF)* (referred to as *myl7:EGFP* and *kdrl:mRPF*, respectively) lines using the Tol2 transposon system (Fig. 1A). EGFP and mRFP expression exhibited a layered pattern in the heart, with mRFP residing within the EGFP expression domain at 96 hpf (Fig. 1B). The atrium and ventricle expressed mRFP and EGFP at 96 hpf (Fig. 1B' and B”). The optical section of the heart clearly supported the layered expression pattern, in which EGFP was detected in the outer layer (myocardial layer) and mRFP in the inner layer (endocardial layer) (Fig. 1C to C”). The two-layered chambers of the atrium and ventricle were separated by the endocardial cushions detected using mRFP (Fig. 1C). The double transgenic line *myl7:EGFP*;*kdrl:mRFP* visualized the blood vessel (endothelium) as well as the heart (myocardium and endocardium) morphology simultaneously (Fig. 1D to G). *kdrl:mRFP* also marked vascular patterning in the craniofacial and trunk regions (Fig. 1E to G). *−6.8kdrl:mRFP* showed increased intensity of the early stage of blood vessel formation at 22 ss (26 hpf) and 1 day post-fertilization (dpf; Fig. S1A to D); however, overall patterning of blood vessel, including cardiac blood vessels, were the same at 4 dpf (Fig. S1E and F). Therefore, our transgenic lines were used for the analysis of chemical-induced heart defects at 96 hpf.

### Live imaging of cardiac development using *myl7:EGFP* and *kdrl:mRFP* line

**Fig. 2.**
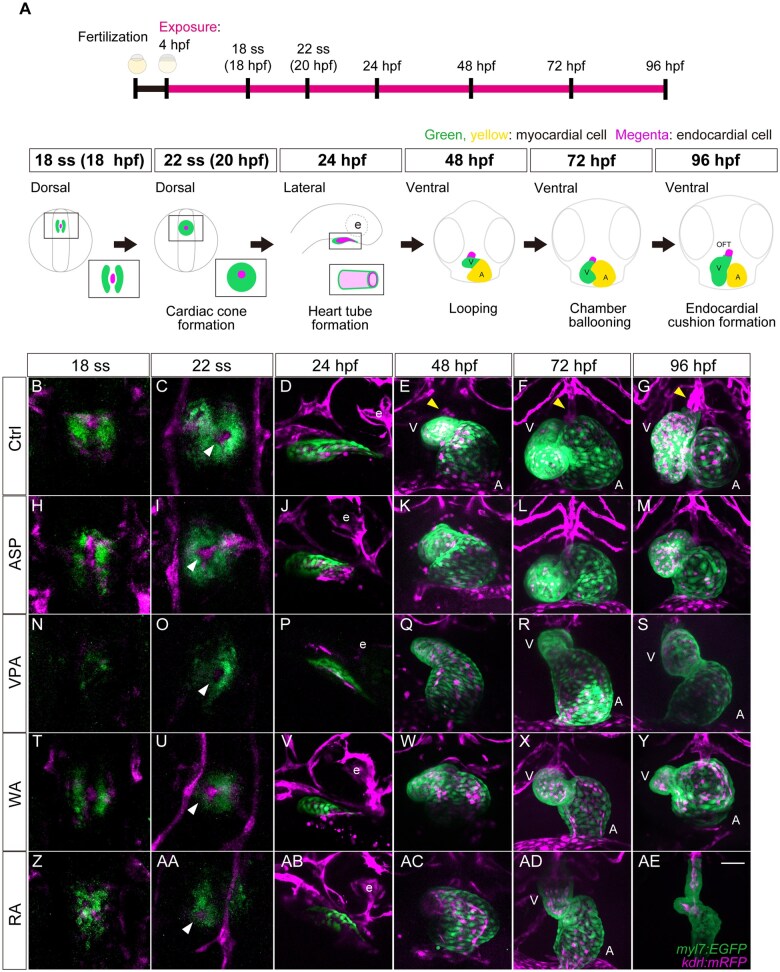
Cardiovascular development and identified defects in teratogen-treated *myl7:EGFP;kdrl:mRFP* zebrafish embryos. (A) Schematic illustration of heart development from 18 somite stage (ss) to 96 hpf. (B to AE) Live imaging of cardiovascular development in control and teratogen-treated zebrafish embryos. Three embryos were used for confocal live imaging. Maximum intensity projection of z-stack images was performed. (B to G) Cardiovascular development in the control embryos. (H to M) Aspirin-treated embryos. (N to S) Valproic acid-treated embryos. (T to Y) Warfarin-treated embryos. (Z to AE) Retinoic acid-treated embryos. The white arrowheads indicate blood vessels in the cardiac cone. The yellow arrowhead indicates the primitive outflow tract. ASP: aspirin; VPA: valproic acid; WA: warfarin; RA: retinoic acid; A: atrium; e: eye; OFT: outflow tract; V: ventricle. Scale bar: 100 µm.

To extend our analysis of heart development, we next focused on the developmental process of the heart (Fig. 2). After cardiac precursors were formed in the anterior lateral plate mesoderm (ALPM), they started migrating toward the midline of the embryo at the 18–20 ss (18 to 19 hpf, Fig. 2A and B). At this stage, EGFP showed bilateral expression, and mRFP was localized between the EGFP expression domains. As heart development proceeded, the migration and subsequent fusion of myocardial precursors formed the cardiac cone at 22 ss (20 hpf), which is called “cardiac fusion” (Fig. 2A and C). In the cardiac cone, the mRFP-positive domain was centrally located within the EGFP domain (Fig. 2C, white arrowhead). Following cardiac fusion, the cone gradually elongated posteriorly, and as the cone extension progressed, the heart primordium started to form a tubular structure, i.e. heart tube formation (Fig. 2A and D). The EGFP-positive myocardium surrounded the mRFP-positive endocardial precursors, i.e. the heart tube. Around this stage, the heart tube gradually started contractile movement. Following the completion of tube formation, the heart tube began to transform into the two-chambered structure consisting of the ventricle and atrium (Fig. 2A and E). At this stage, the heart tube also started looping (cardiac looping), and the atrioventricular boundary became evident. The primitive outflow tract (OFT) was also observed (Fig. 2E, yellow arrowhead). As cardiac looping and ballooning progressed, the heart tube formed an S-shaped structure with left–right asymmetry (Fig. 2A and E). As the primitive heart started growth, the characteristic cardiomyocyte morphology became more evident by 72 hpf (Fig. 2A and F). The OFT also increased in size at approximately this stage (Fig. 2F, yellow arrowhead). By 96 hpf, the ventricle and atrium had increased in size, and the endocardial cushions were formed at the boundary between the atrium and ventricle (Figs 2A and G and 7A). Around this stage, cardiac trabeculation proceeded, creating the sponge-like muscular structure (trabecula) lined by the endocardial layer (Figs 2G and 7B to B”). Thus, our established *myl7:EGFP* and *kdrl:mRFP* transgenic zebrafish lines depicted the heart morphology (myocardium and endocardium) during embryonic cardiogenesis.

### Teratogen-induced heart defects from the progenitor to tube formation stages

Next, we examined the chemical-induced heart defects in zebrafish embryos utilizing transgenic lines. The following 4 teratogens were used in this study: ASP, VPA, WA, and RA (Table 1). These teratogens are known to cause heart defects in mammals (Taylor et al. 1980; Lammer et al. 1985; Ardinger et al. 1988; Gupta et al. 2003; Alsdorf and Wyszynski 2005; Starling et al. 2012; D’Souza et al. 2017). The zebrafish embryos were treated with the chemicals at 4 hpf (sphere stage during the blastula period), and their heart morphology was analyzed chronologically (Fig. 2A to AE).

As described above, in the control embryos, EGFP was bilaterally expressed with mRFP at 18 ss prior to cardiac fusion (Fig. 2B). The intensity of bilateral EGFP expression and the size of the expression domain decreased in VPA-, WA-, and RA-treated embryos (Fig. 2B, N, T, and Z), whereas ASP showed significantly less effect (Fig. 2B and H).

**Fig. 3.**
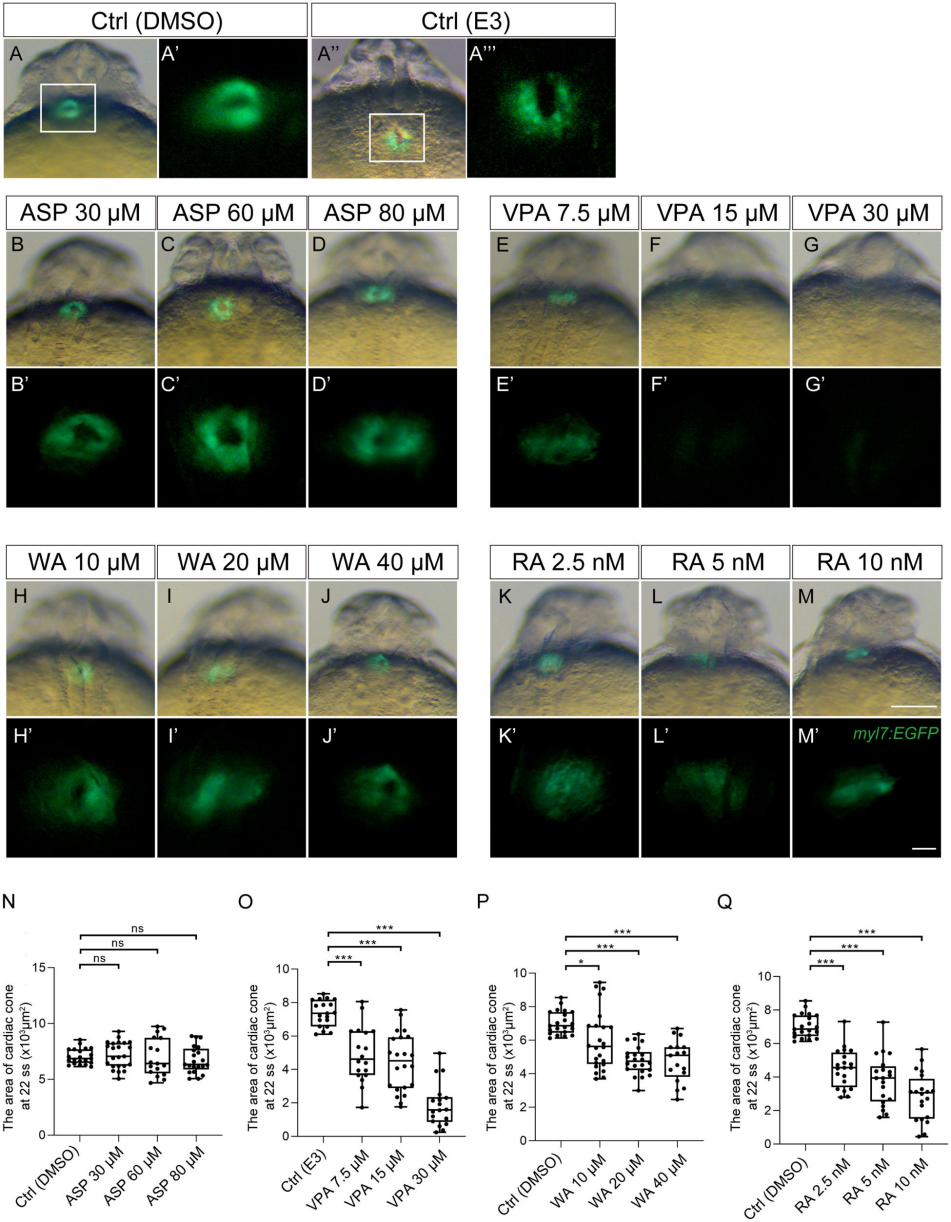
Quantification of cardiac cone formation. Measurement of the cardiac cone area at 22 ss after teratogen exposure was performed. (A to M, A") DMSO (A) and E3 (A") were used as control treatment. Effect of ASP (B to D), VPA (E to G), WA (H to J), and RA (K to M) treatment on cardiac cone formation. (A' to M', A'") A magnified view of the region indicated by the rectangle in panel A. (N to Q) Quantification of the area of cardiac cone at 22 ss. Control (Ctrl; DMSO): *n* = 21; Ctrl (E3) *n* = 19; ASP (30, 60, 80 µM): *n* = 23, 17, 21; VPA (7.5, 15, 30 µM): *n* = 18, 22, 18; WA (10, 20, 40 µM): *n* = 24, 22, 17; RA (2.5, 5, 10 nM): *n* = 20, 20, 20. *P < 0.05, ***P < 0.001 (one-way ANOVA followed by Dunnett’s multiple comparison test). Scale bars: 500 µm in A to M; 100 µm in A' to M'.

In control embryos, the cardiac corn was formed at 22 ss, in which the mRFP expression domain (endocardium) was surrounded by EGFP (myocardium) (Fig. 2C, white arrowhead). In the teratogen-treated embryos, though the cardiac cone was formed, its size and morphology were affected by VPA, WA, and RA (Fig. 2C, O, U, and AA). The 3 teratogens (VPA, WA, and RA) also reduced EGFP expression. In particular, VPA treatment greatly reduced mRFP expression, whereas the effects of WA and RA were minimal, suggesting that VPA severely impaired blood vessel formation at this stage (Fig. 2C, O, U, and AA). Overall, ASP treatment resulted in a minor decrease in both EGFP and mRFP expression (Fig. 2C and I). These results were confirmed by quantifying myocardial defects in a dose-dependent manner (Fig. 3). ASP treatment did not induce a statistically significant difference in the cardiac cone area (Fig. 3A to 3D' and N). However, VPA, WA, and RA treatments resulted in a significant decrease in the cardiac cone area (Fig. 3A to A'”, E to M', and O to Q).

**Fig. 4.**
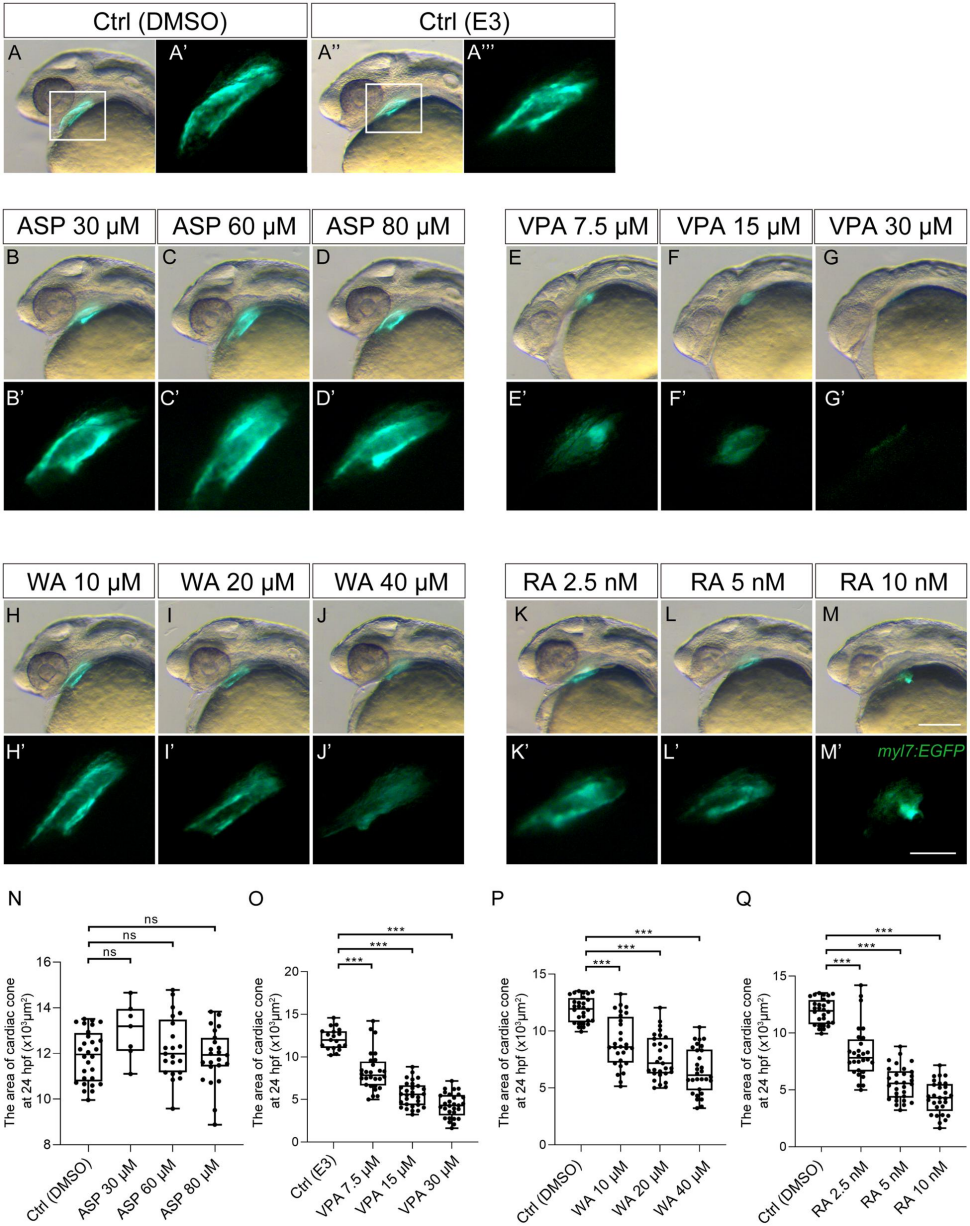
Quantification of heart tube formation. Measurement of heart tube length at 24 hpf after teratogen exposure was performed. (A to M, A") DMSO (A) and E3 (A") were used as control treatment. Effect of ASP (B to D), VPA (E to G), WA (H to J), and RA (K to M) treatment on heart tube formation. (A' to M') A magnified view of the region indicated by the rectangle in panel A. (N to Q) Quantification of the heart tube area. Control (Ctrl; DMSO): *n* = 28; Ctrl (E3) *n* = 19; ASP (30, 60, 80 µM): *n* = 7, 21, 23; VPA (7.5, 15, 30 µM): *n* = 28, 30, 29; WA (10, 20, 40 µM): *n* = 28, 29, 30; RA (2.5, 5, 10 nM): *n* = 28, 29, 30. ***P < 0.001 (one-way ANOVA followed by Dunnett’s multiple comparison test). Scale bars: 200 µm in A to M; 50 µm in A' to M'.

At the tube formation stage (24 hpf), the endocardial layer was surrounded by the myocardial layer (Fig. 2D). During teratogen exposure, the heart tube was formed, but the size of the heart tube was smaller than that of the control group in the VPA, WA, and RA-treated embryos (Fig. 2D, P, V, and AB). Notably, VPA-treated embryos displayed the most severe defects in vasculogenesis at 22 ss, but blood vessel formation tended to catch up at 24 hpf (Fig. 2D and P). By contrast, ASP treatment did not show a significant decrease EGFP or mRFP expression (Fig. 2D and J). Impairment of heart tube formation was also quantified (Fig. 4). ASP treatment did not cause a significant decrease in the heart tube area (Fig. 4A to D' and N). However, the heart tube area significantly decreased in a dose-dependent manner in VPA-, WA-, and RA-treated embryos (Fig. 4A to A'”, E to M', and O to Q). Therefore, the major stages of heart development, including cardiac fusion and tube formation, anyhow proceeded during teratogen treatment; however, the overall structure was smaller compared with the control (except for ASP).

### Teratogen-induced heart defects from the cardiac looping to chamber formation stages

**Fig. 5.**
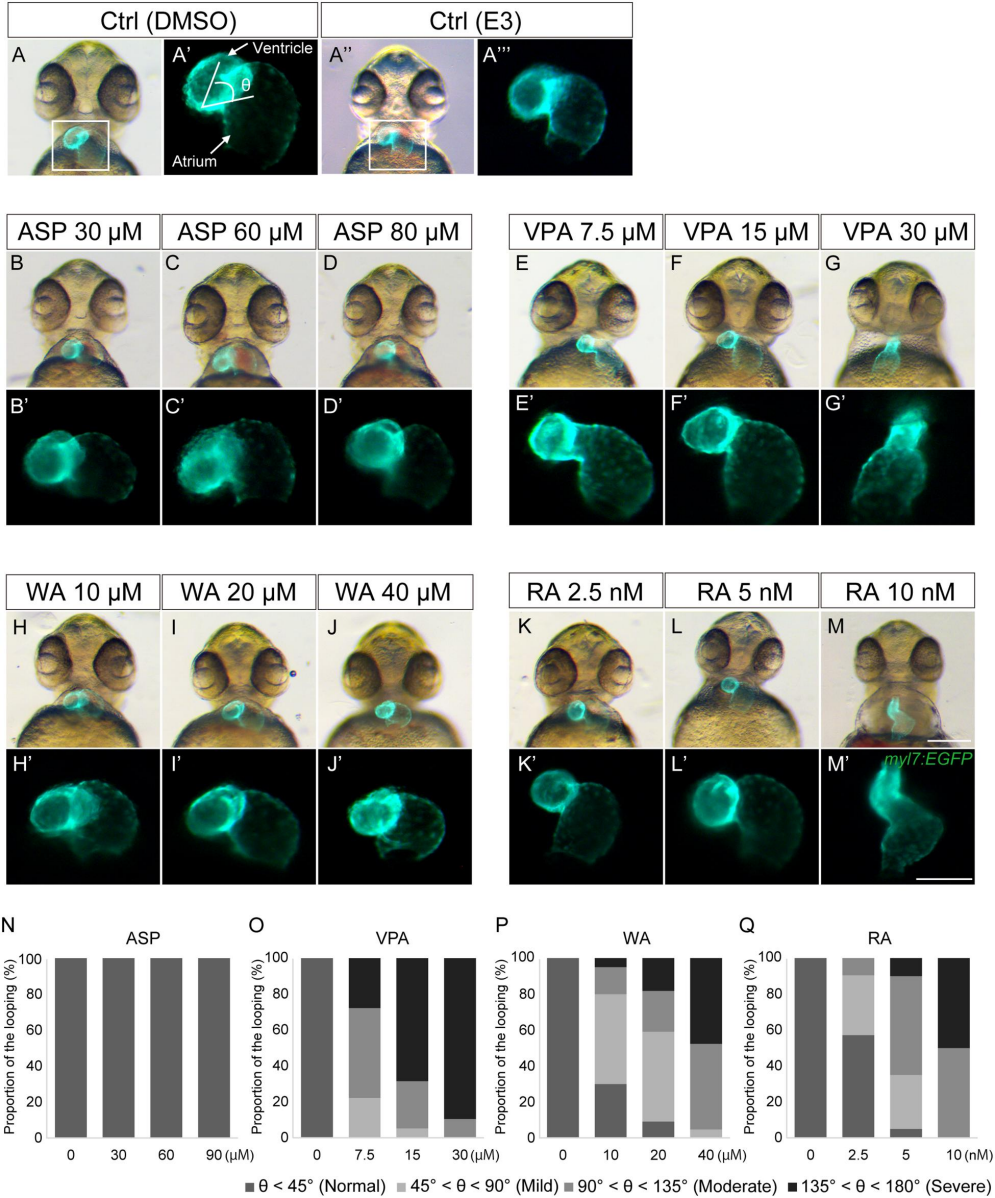
Quantification of cardiac looping. (A to M and A") DMSO (A) and E3 (A") were used as control treatment. Effect of ASP (B to D), VPA (E to G), WA (H to J), and RA (K to M) treatment on cardiac looping at 48 hpf. (A' to M' and A'") A magnified view of the region indicated by the rectangle in panel A. (N to Q) Quantification of cardiac looping. To quantify cardiac looping, the angle between the ventricle and atrium was measured at 48 hpf (A'), following Chen et al. 2019 and Zhang et al. 2021. According to severity of cardiac looping, the states were classified into normal (θ < 45°), mild (45° < θ < 90°), moderate (90° < θ < 135°), and severe (135° < θ < 180°). Control (Ctrl; DMSO): *n* = 21; Ctrl (E3) *n* = 21; ASP (30, 60, 80 µM): *n* = 17, 18, 19; VPA (7.5, 15, 30 µM): *n* = 19, 19, 19; WA (10, 20, 40 µM): *n* = 20, 22, 21; RA (2.5, 5, 10 nM): *n* = 21, 20, 20. Scale bars: 200 µm in A to M; 50 µm in A' to M'.

**Fig. 6.**
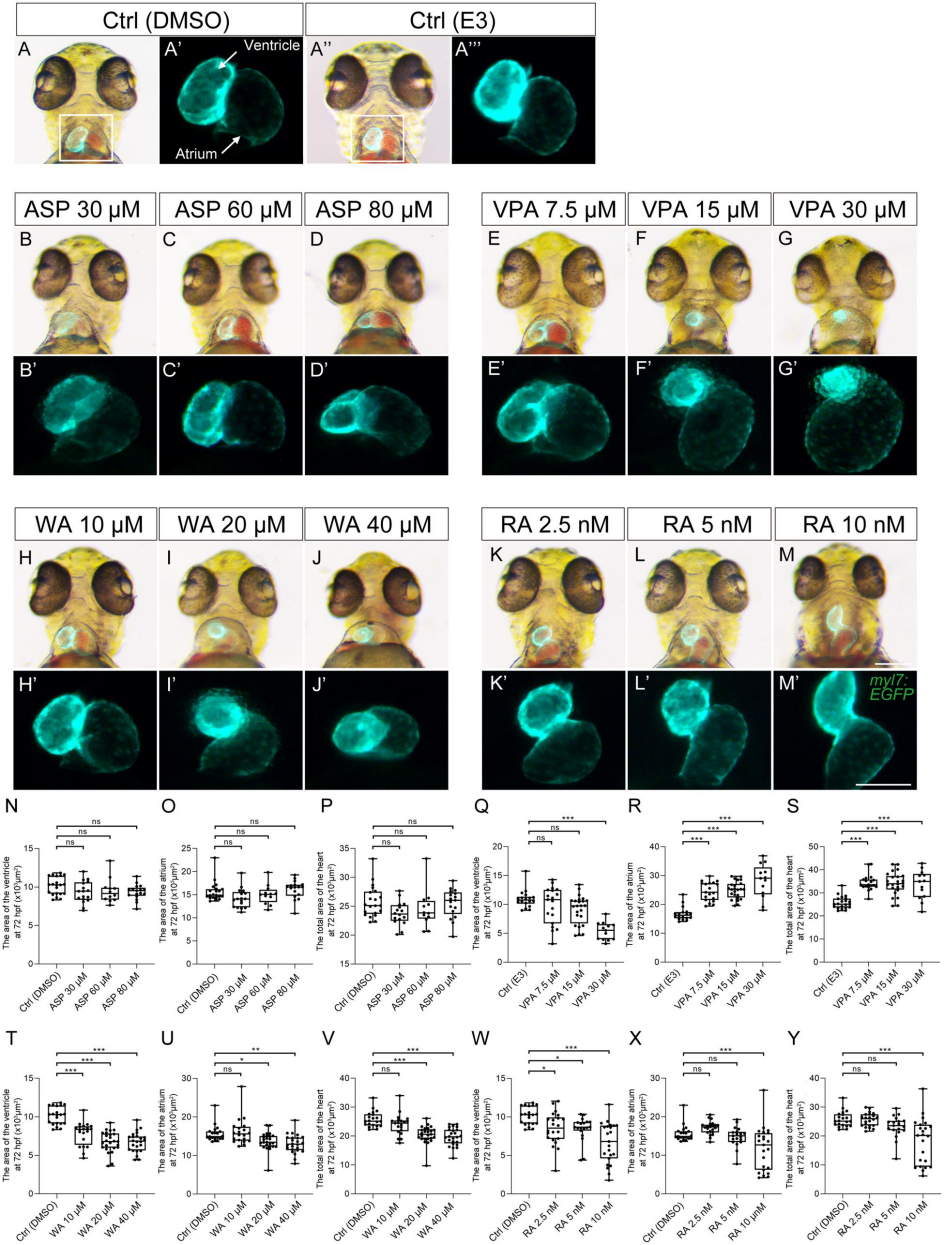
Quantification of cardiac ballooning. To quantify cardiac ballooning, the areas of the ventricle, atrium, and the total area of both were measured at 72 hpf after teratogen exposure. (A to M and A") DMSO (A) and E3 (A") were used as control treatment. Effect of ASP (B to D), VPA (E to G), WA (H to J), and RA (K to M) treatment on cardiac ballooning. (A' to M' and A'") A magnified view of the region indicated by the rectangle in panel A. (N to Y) Quantification of the area of the ventricle (N, Q, T, and W), atrium (O, R, U, and X), and the total area of the ventricle and atrium (P, S, V, and Y) was performed. Control (Ctrl; DMSO): *n* = 21; Ctrl (E3) *n* = 18; ASP (30, 60, 80 µM): *n* = 17, 12, and 17; VPA (7.5, 15, and 30 µM): *n* = 19, 24, and 13; WA (10, 20, and 40 µM): *n* = 20, 25, and 24; RA (2.5, 5, and 10 nM): *n* = 22, 19, and 23. *P < 0.05, **P < 0.01, ***P < 0.001 (one-way ANOVA followed by Dunnett’s multiple comparison test). Scale bars: 200 µm in A to M; 50 µm in A' to M'.

The heart tube started looping and chamber formation by 48 hpf (Fig. 2E). The VPA-, WA-, and RA-treated embryos showed minor defects in cardiac looping (Fig. 2E, Q, W, and AC). ASP-treated embryos showed no distinct morphological difference compared with the control embryos (Fig. 2E and K). Defects in cardiac looping were quantified by measuring the angle between the ventricle and atrium (Fig. 5A and A'; Chen et al. 2019; Zhang et al. 2021). In ASP-treated embryos, cardiac looping was not inhibited (Fig. 5A to D' and N). However, looping defects were observed in a dose-dependent manner in VPA-, WA-, and RA-treated embryos (Fig. 5A to A'”, E to M', and O to Q). The ventricle and atrium were formed in VPA-, WA-, and RA-treated embryos, along with primitive OFT, albeit with abnormal morphology (Fig. 2E, Q, W, and AC). By 72 hpf, chamber ballooning was completed, and 2 distinct chambers, the ventricle and atrium were clearly observed in the control group (Fig. 2F). Also in the control group, cardiac chambers increased in volume, and outer and inner curvatures were observed. In VPA-, WA-, and RA-treated embryos, heart looping and the morphogenesis of the ventricle and atrium were severely impaired (Fig. 2F, R, X and AD). VPA-treated embryos displayed a reduction in the size of the ventricle and a dilated atrium (Fig. 2F and R). The tendency was statistically significant in the reduction of ventricle size and dilation of the atrium (Fig. 6A”, A'”, E to G', Q, and R). Additionally, the total heart area was significantly increased in a dose-dependent manner, suggesting proportional defects (Fig. 6S). These embryos also failed to complete heart looping. The looping progressed in WA-treated embryos, but was not complete at this stage (Fig. 2F and X). WA-treated embryos showed a significant decrease in ventricle and atrium size in a dose-dependent manner (Fig. 6A, A', H to J', and T to V). RA-treated embryos also showed incomplete cardiac looping and abnormal morphology of the ventricle and atrium (Figs 2F and AD and 6A, A', and K to M'). The size of the ventricle and atrium significantly decreased in a dose-dependent manner (Fig. 6W to Y). The surface of the ventricle and atrium was smooth in control embryos, but rough in RA-treated embryos (Fig. 2F and AD). The OFT was formed in teratogen-treated embryos, but abnormal in morphology (except for ASP). Essentially, no looping or chamber phenotypes were observed in ASP-treated embryos (Figs 2F and L and 6A to D' and N to P).

**Fig. 7.**
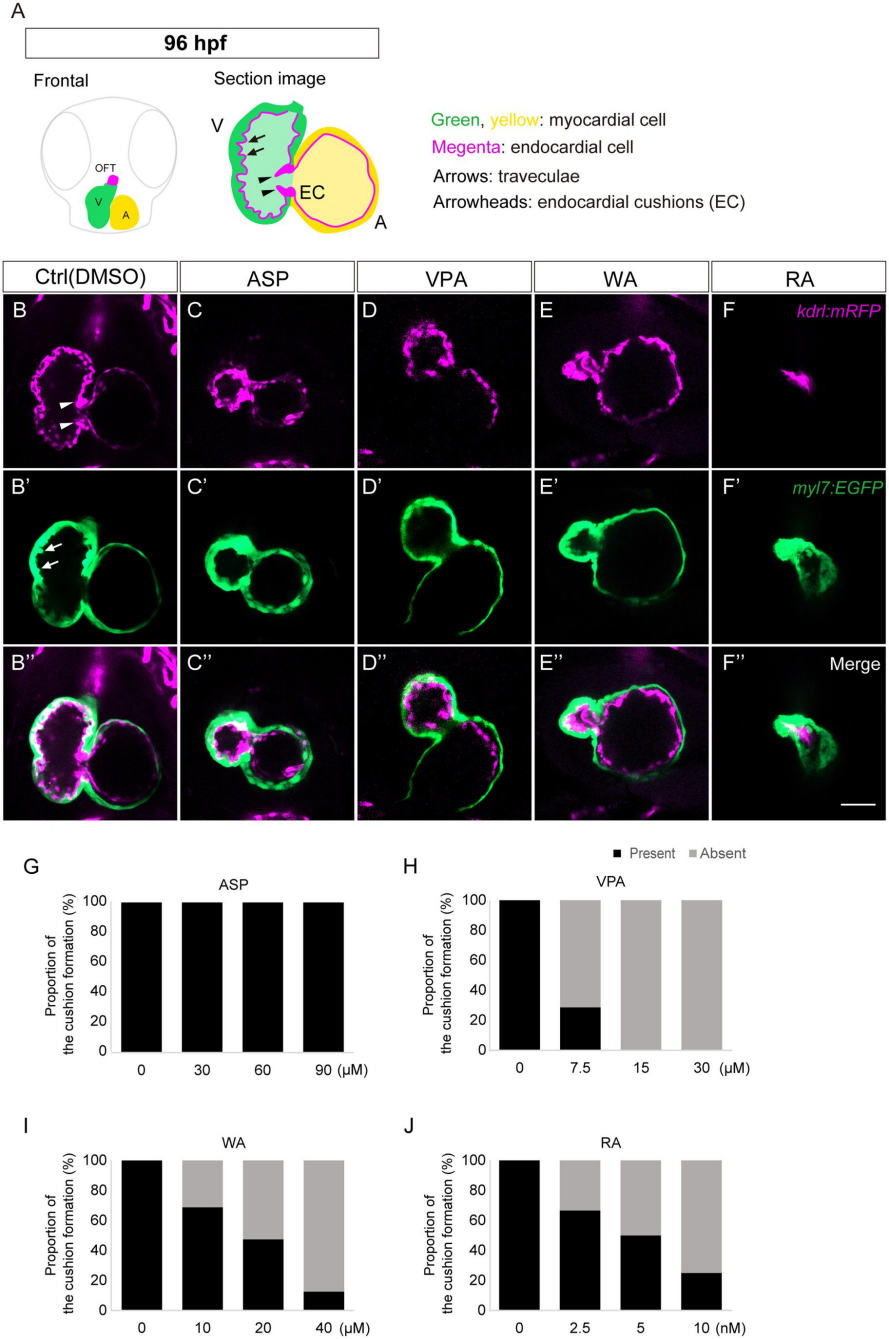
Optical section of zebrafish heart at 96 hpf. (A) Schematic illustration of the heart and its section image. Zebrafish embryos were treated with teratogens. Optical sections at 96 hpf were obtained using confocal microscopy for control (B to B"), ASP (C to C"), VPA (D to D"), WA (E to E"), and RA (F to F"). (G to J) Quantification of endocardial cushion formation. The presence of the endocardial cushions at 96 hpf was evaluated in ASP (G), VPA (H), WA (I), and RA (J) in a dose-dependent manner. Control (Ctrl): *n* = 17; ASP (30, 60, 80 µM): *n* = 11, 12, 12; VPA (7.5, 15, 30 µM): *n* = 7, 11, 12; WA (10, 20, 40 µM): *n* = 16, 19, 8; RA (2.5, 5, 10 nM): *n* = 15, 10, 12. Arrowheads indicate the endocardial cushions (EC). Arrows indicate the trabeculae. A: the atrium; V: the ventricle. Scale bar: 100 µm.

At 96 hpf, embryonic heart morphogenesis was nearly complete in the control embryos, including the chambers (ventricle and atrium), OFT, trabeculae, and endocardial cushions (Figs 2G and 7A and B to B”). VPA-treated embryos still showed incomplete looping and underdevelopment of the ventricle and atrium (Fig. 2G and S). The ventricular wall was thinner than that of the control, and neither the trabeculae nor the endocardial cushions were formed in VPA-treated embryos (Fig. 7B to B” and D to D”). Endocardial cushions were not formed in a dose-dependent manner in VPA-treated embryos (Fig. 7H). WA-treated embryos also showed incomplete looping, a smaller ventricle, and a dilated atrium (Fig. 2G and Y). Additionally, endocardial cushions were not formed in dose-dependent manner in WA-treated embryos, and trabeculae formation was impaired (Fig. 7B to B” and E to E”, and I). RA-treated embryos exhibited a string-like morphology, in which the ventricle and atrium were thinly elongated with the rough surface (Fig. 2G and AE). The size of the ventricle and atrium in RA-treated embryos was notably smaller than that of the control (Fig. 7B to B” and F to F”). Additionally, endocardial cushions were not formed in a dose-dependent manner in the string-like heart (Fig. 7J). Again, no defect in heart morphogenesis was observed in ASP-treated embryos (Figs 2G and M and 7B to B”, C to C”, and G).

Taken together, zebrafish embryos were susceptible to the known teratogens tested. In teratogen-treated embryos, the cardiac cone and tube were formed, although their size was smaller. At the later stages of heart development, looping and chamber formation were severely affected. The heart size of the teratogen-treated embryos was eventually smaller than that of the controls.

### Disruption of cardiac progenitors following teratogen exposure

**Fig. 8.**
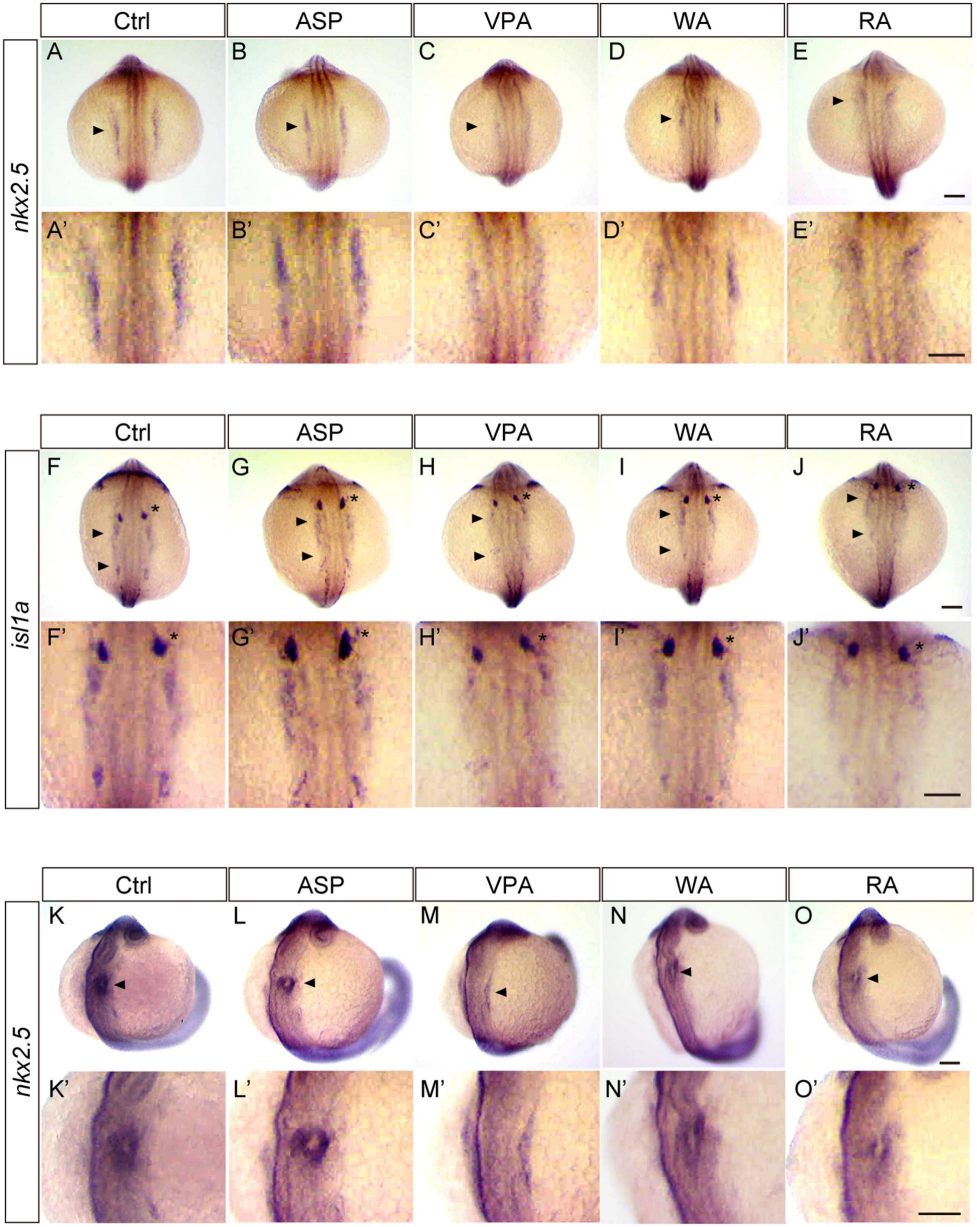
Expression of cardiac progenitor marker in teratogen-treated embryos. (A to E) Expression domain of *nkx2.5* in teratogen-treated zebrafish embryos at 13 somite stage (ss, 15.5 hpf). The expression of *nkx2.5* was detected bilaterally at the dorsal side, which is the anterior lateral plate mesoderm (ALPM). The expression was severely decreased following teratogen exposure, except for ASP exposure. The arrowhead indicates *nkx2.5* expression domain. (A' to E') Magnified view of panels A to E. (F to J) Expression domain of *isl1a* in teratogen-treated zebrafish embryos at 13 ss. The expression was also detected in the ALPM; however, the expression was severely decreased following teratogen exposure, except for ASP exposure. The arrowheads indicate *isl1a* expression domain. The asterisk indicates the expression of trigeminal placode and was not affected by teratogen exposure. (F' to J') Magnified view of panels F to J. (K to O) Expression domain of *nkx2.5* in teratogen-treated zebrafish embryos at 20 ss. The expression was detected in the cardiac cone (arrowhead). The expression was reduced following teratogen exposure, except for ASP exposure. (K' to O') Magnified view of panels K to O. The sample size used for whole-mount in situ hybridization was 30 for Ctrl, ASP, VPA, WA, and RA. Scale bars: 100 µm.

The above results suggested that teratogens affected the development of cardiac progenitors at earlier stages. To address this, we examined the expression of the homeobox transcription factor *nkx2.5* and LIM homeobox transcription factor *islet1a* (*isl1a*) by whole-mount in situ hybridization (Fig. 8). These genes are known as cardiac progenitor markers among vertebrates, and their expression is detected earlier than that of EGFP and mRFP in our Tg lines (Chen and Fishman 1996; Cai et al. 2003; Hami et al. 2011; Witzel et al. 2012). Zebrafish embryos were treated with the teratogens and fixed at 13 ss (15.5 hpf). For *nkx2.5*, the signal was bilaterally detected in the second heart field of the control embryos (Fig. 8A and A').

The expression of *nkx2.5* was not affected by ASP treatment (Fig. 8A, A', B, and B') but was severely reduced in VPA-treated embryos (Fig. 8A, A', C, and C'). In WA- and RA-treated embryos, *nkx2.5* expression was also decreased and became diminished toward the posterior (Fig. 8A, A', D, D', E, and E'). *isl1a* was also bilaterally expressed in the ALPM of control embryos (Fig. 8F and F'). *isl1a* expression was not affected by ASP treatment (Fig. 8F, F', G, and G') but was severely decreased in VPA- and RA-treated embryos (Fig. 8F, F', H, H', J, and J'). WA-treated embryos also showed decreased *isl1a* expression (Fig. 8F, F', I, and I'). Thus, these results demonstrate that teratogen exposure impairs the development of cardiac progenitors.

At the later stage (20 ss, 19 hpf), cells expressing *nkx2.5* migrated to form the cardiac cone, and the expression domain of *nkx2.5* became donut-shaped (Fig. 8K and K'). In WA- and RA-treated embryos, *nkx2.5*-expressing cells were present in the cardiac cone, but the expression was abnormal. The expression level was reduced, and the donut shape was disorganized (Fig. 8K, K', N, N', O, and O'). Notably, VPA-treated embryos maintained bilateral expression of *nkx2.5* in the ALPM (Fig. 8K, K', M, and M'). Therefore, VPA, WA, and RA all affected the migration of cardiac progenitors, and among them, VPA exhibited the strongest effect. ASP exposure did not affect the expression domain of *nkx2.5* in the cardiac cone (Fig. 8K, K', L, and L'). These results presented above suggest the existence of a critical period, which is a specific developmental window when the heart is highly susceptible to teratogen.

### The critical period of heart defects

**Fig. 9.**
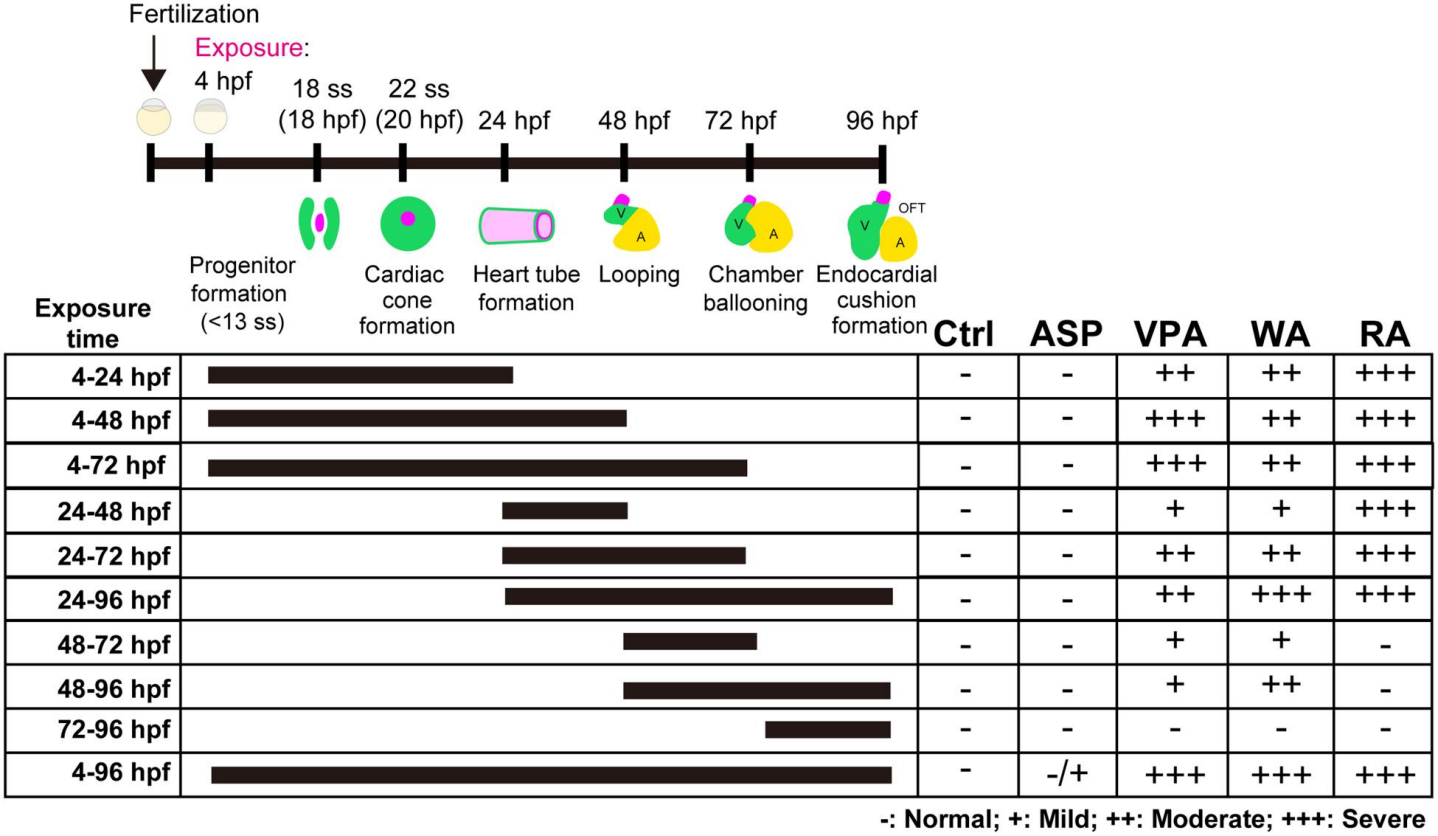
Critical period for heart defects in zebrafish embryos. Zebrafish embryos were exposed to teratogens for 10 distinct exposure windows: 4 to 24, 4 to 48, 4 to 72, 24 to 48, 24 to 72, 24 to 96, 48 to 72, 48 to 96, 72 to 96, and 4 to 96 hpf. Heart defects were assessed at 96 hpf following teratogen exposure.

Next, we examined the critical period for inducing heart malformations (Fig. 9). Zebrafish embryos were treated with the teratogens for 10 distinct exposure periods as follows: 4 to 24, 4 to 48, 4 to 72, 24 to 48, 24 to 72, 24 to 96, 48 to 72, 48 to 96, 72 to 96, and 4 to 96 hpf. In this analysis, we determined which exposure period sufficiently caused major cardiac malformations, similar to that of each 4 to 96 hpf exposure group (Fig. 9). For 3 teratogens (VPA, WA, and RA) that strongly affected cardiac morphogenesis, no malformation was observed after 72 to 96 hpf exposure, indicating that the critical period for the 3 teratogens resides 4 to 72 hpf.

For VPA, major malformations were induced by 4 to 48 hpf exposure, whereas the effects were moderate after 4 to 24, 24 to 72, and 24 to 96 hpf exposures, indicating that the 4 to 48 hpf is the most critical period for VPA. For WA, the critical period could be a rather broad, ranging from 4 to 72 hpf, as major malformations were only obtained at 24 to 96 hpf (in addition to the full exposure period of 4 to 96 hpf). The shortest exposure period that induced a moderate phenotype was 4 to 24 hpf. For RA, major malformations were equally induced after 4 to 24, 4 to 48, 24 to 48, 24 to 72, and 24 to 96 hpf exposures, and no major malformations were observed after following 48 to 72, 48 to 96, and 72 to 96 hpf exposures, indicating that the exposure time before 48 hpf are the most important for RA. Overall, although each teratogen has a unique window, the 4 to 48 hpf is shared by all 3 as a sensitive period.

### Heartbeat defect of the teratogens

Cardiac malformations are often accompanied by cardiac functional defects, such as arrhythmias. Thus, we established a transgenic line to analyze heart function in which the blood flow was monitored (Fig. S2). For this, we selected the transcriptional factor *gata1* as a marker, specifically expressed in blood cells, and generated *Tg(−8.0gata1:mKate2)* (referred to as *gata1:mKate2*) lines using the Tol2 transposon system (Fig. S2). The double transgenic line, *myl7:EGFP; gata1:mKate2*, was treated with VPA, WA, and MTX at 4 hpf and subjected to live imaging of the heart at 2 dpf using a light sheet microscope (Movies S1 to S4). We chose these 3 teratogens because they are known to cause cardiotoxicity and heart malformations in mammals (Fuskevag et al. 2000; Perez-Verdia et al. 2005). Using the double transgenic line, we monitored blood flow by visualizing mKate-labeled blood cells and heart malformation by EGFP-labeled cardiomyocytes.

At this stage (2 dpf), cardiac looping and chamber formation (2 distinct cardiac chambers: the ventricle and atrium) were underway (Fig. 2A). In the control embryos, regular heartbeats and blood flow were visualized as green signal (driven by the *myl7* promoter) and magenta signal (driven by the *gata1* promoter), respectively (Movie S1). The atrioventricular boundary was distinct and regular heart contractions were also observed (Movie S1). The VPA-treated embryos exhibited an elongated heart (indicating a looping defect) with less distinct atrioventricular boundary (Movie S2). This malformation was found to be accompanied by severe loss of rhythmic chamber contraction (Movie S2). In WA-treated embryos, looping was defective, together with a loss of rhythmic chamber contraction, although the atrioventricular boundary was formed (Movie S3). MTX-treated embryos showed looping defects and loss of rhythmic chamber contraction, with the normal formation of the atrioventricular boundary. Furthermore, the ventricle exhibited abnormal movement (Movie S4). Defects in blood flow, such as heart failure, were observed in all the teratogen-treated embryos. Therefore, using our transgenic lines, we were able to assess both the function and morphology of the embryonic heart.

## Discussion

In the present study, we established transgenic lines, *myl7: EGFP* and *kdrl: mRFP*, to visualize heart development and analyze the disturbance of heart development leading to heart morphological defects. Unlike previous studies (Dyballa et al. 2019; Chen et al. 2024; Lei et al. 2024), we focused on developmental processes, and through this approach, we determined the critical periods for each teratogen in fish. Furthermore, we established a transgenic line, *gata1: mKate2*, to monitor blood flow in the heart because heart malformations are often associated with functional defects, such as arrhythmia. The transgenic embryos were treated with VPA, WA, RA, and ASP. These teratogens, except ASP, commonly impaired the development of cardiac progenitors, leading to hypoplasia of both the heart cone and heart tube. These embryos exhibited abnormal looping that ultimately resulted in heart malformations, including those in the chambers and endocardial cushions. These results are consistent with previous reports on mammals (Taylor et al. 1980; Lammer et al. 1985; Ardinger et al. 1988; Alsdorf and Wyszynski 2005; Starling et al. 2012; D’Souza et al. 2017). Additionally, based on meta-analysis and epidemiological studies, ASP exposure during pregnancy does not induce heart defects in human (Werler et al. 1989; Kozer et al. 2002). However, ASP are reported to induce heart defects in rat (Gupta et al. 2003). In our study, ASP did not cause any structural heart defects, but a transient developmental delay was observed, which resolved spontaneously and is not considered teratogenic. This evidence suggests that zebrafish embryos could be a reliable model to evaluate potential causing heart defects in human. Thus, our established transgenic lines could be a suitable tool for analyzing the mechanisms underlying chemical-induced cardiac malformations in humans. Furthermore, the *gata1*-reporter line allows us to assess heart beats and defects, together with structural abnormalities of the heart. Taken together, the combination of these 3 transgenic lines would provide a versatile platform to evaluate heart defects and cardiotoxicity caused by chemical agents, from morphology to function.

We administered different teratogens (ASP, VPA, WA, and RA) to zebrafish embryos and identified 2 critical developmental events that are sensitive to teratogen exposure: The first event is progenitor formation and the other is cardiac looping. In our study, we selected *nkx2.5* and *isl1a* as universal cardiac progenitor markers to assess cardiac progenitor formation. The expression levels of *nkx2.5* and *isl1a* were decreased following exposure to VPA, WA, and RA in zebrafish embryos. Furthermore, their expression domains decreased in size. These observations indicate that chemical exposure affects the number and/or the specification of cardiac progenitor cells. Despite this, subsequent development, such as heart cone and heart tube formation (20 to 24 hpf), did occur, albeit at a smaller size compared with the controls. This observation is consistent with previous findings by Keegan et al. (2005), suggesting that the number of cardiac progenitors, once reduced, is hard to restore during later development. This disturbance could have greater impacts on later morphogenesis of the heart, and thus, progenitor formation is a susceptible step for teratogen-induced heart malformations. The reduction in the number of cardiac progenitors and the resulting smaller heart cone and heart tube were common features in all chemical-treated embryos, except for those treated with ASP. Additionally, we observed defects in cardiac looping, a crucial step in heart morphogenesis starting at 35 to 48 hpf, in embryos exposed to all 3 chemicals (VPA, WA, and RA). Despite common progenitor defects, abnormalities in cardiac looping varied among the teratogens. Because teratogen treatment, excluding the period of progenitor formation (i.e. treatment of 24 to 96 hpf), still severely affected heart morphogenesis, including looping, it is reasonable to conclude that heart looping is also a susceptible step for teratogen-induced heart malformations. Notably, the critical period of 4 to 48 hpf in zebrafish corresponds to E7.5 to E9.5 in mice and days 18–28 in humans (Mahler and Butcher 2011; Krishnan et al. 2014). Probably due to defects in progenitor formation and/or looping, the morphogenesis of the chamber and OFT showed abnormalities, such as reduced size (ventricle and OFT) and dilatation (atrium), depending on the teratogen used.

VPA, WA, and RA similarly affected early cardiac progenitor cell formation, but their effects varied during looping and chamber formation. For example, RA-treated embryos showed severe looping defects, string-like morphology (elongated ventricle and atrium), and a rough surface of the heart. These differences in morphological outcomes could reflect distinct mechanisms by which each teratogen impairs heart development.

Zebrafish mutants isolated by ENU screening provide some insights into the molecular mechanisms underlying teratogen-induced cardiac malformations (Chen et al. 1996; Stainier et al. 1996). For instance, mutants such as, *acerebellar* (*fgf8*, Reifers et al. 2000) and *weak atrium* (*myh6*; Berdougo et al. 2003) exhibit reduced ventricle size, whereas *santa* (CCM1; Mably et al. 2006) and *valentine* (CCM2; Mably et al. 2006) show ventricular wall thickening. Additionally, *jekyll* (*ugdh*, Walsh and Stainier 2001) mutants present with atrioventricular valve failure. These phenotypes resemble the abnormalities induced by VPA and WA in our study. *heartstrings* (*tbx5*, Garrity et al. 2002) mutants represent a string-like morphology of the heart, which was observed in the RA-treated embryos. These causative genes and related pathways could be the targets of these teratogens. Our results, combined with the existing knowledge from zebrafish mutants and our identified critical periods, we can more rapidly elucidate the mechanisms underlying chemical-induced heart defects.

We have previously used transgenic zebrafish to elucidate the adverse outcome pathway (AOP) related to craniofacial malformations (Liu et al. 2023). Similarly, the transgenic zebrafish models developed in this study can clarify the heart development processes and contribute further to the understanding of the AOP in heart malformations. Our approach will facilitate the elucidation of the intricate mechanisms through which teratogens influence heart development.

The use of the transgenic zebrafish lines *myl7: EGFP* and *kdrl: mRFP* developed in this study presents significant advantages for evaluating cardiac and vascular defects compared with traditional wild-type zebrafish models. Previous studies assessing heart or vascular defects, including those involving the endocardium, have typically used living wild-type zebrafish embryos without labeling any tissue components (Brannen et al. 2010; Selderslaghs et al. 2012; Augustine-Rauch et al. 2016; Inoue et al. 2016). Although wild-type zebrafish comply with the principle of the 3Rs and enable high-throughput teratogenicity screening, their utility remains limited due to the poor visibility of the heart and vasculature, which impedes precise morphological assessments. In the case of the heart, the identification of vascular and endocardial abnormalities in wild-type zebrafish was particularly challenging. Double-transgenic embryos, obtained by crossing *myl7: EGFP* and *kdrl: mRFP* lines, allow us to perform the real-time analysis of heart morphogenesis, including endocardial and vascular formation, through live imaging after the formation of cardiac progenitor cells. This system provides a more accurate and high-throughput screening platform than that provided by wild-type zebrafish.

In addition to the morphological analysis, it is crucial to evaluate heart function such as blood flow, to fully understand cardiotoxicity. Cardiotoxicity has been one of the important indexes in the dropout of drug candidates during the development process (Cook et al. 2014; Pang et al. 2019). Our transgenic zebrafish line *gata1: mKate2* facilitates the assessment of blood flow within the heart and the systemic circulation. Therefore, by crossing this line with the *myl7: EGFP* or *kdrl: MRFP* transgenic lines, we are able to simultaneously evaluate abnormalities in both the morphology and function of the heart and vasculature. This system provides a unique platform for the comprehensive evaluation of the effects of chemical exposures.

In summary, we successfully demonstrated the proof-of-concept of our transgenic systems using 4 teratogenic chemicals: VPA, WA, RA, and ASP, all of which have been reported to cause heart defects in mammals. Future studies should focus on testing a broader variety of chemicals with different physicochemical properties and structures. These efforts will strengthen our system as a reliable alternative to conventional animal models in developmental toxicity assays and research.

## Supplementary Material

kfaf083_Supplementary_Data
