# Supplementary figures and images for: Chemical-induced heart defects using a transgenic zebrafish model
Source: Toxicol Sci. 2025 Jun 12;207(1):57–73. doi: 10.1093/toxsci/kfaf083 (PMC12448204; doi:10.1093/toxsci/kfaf083)

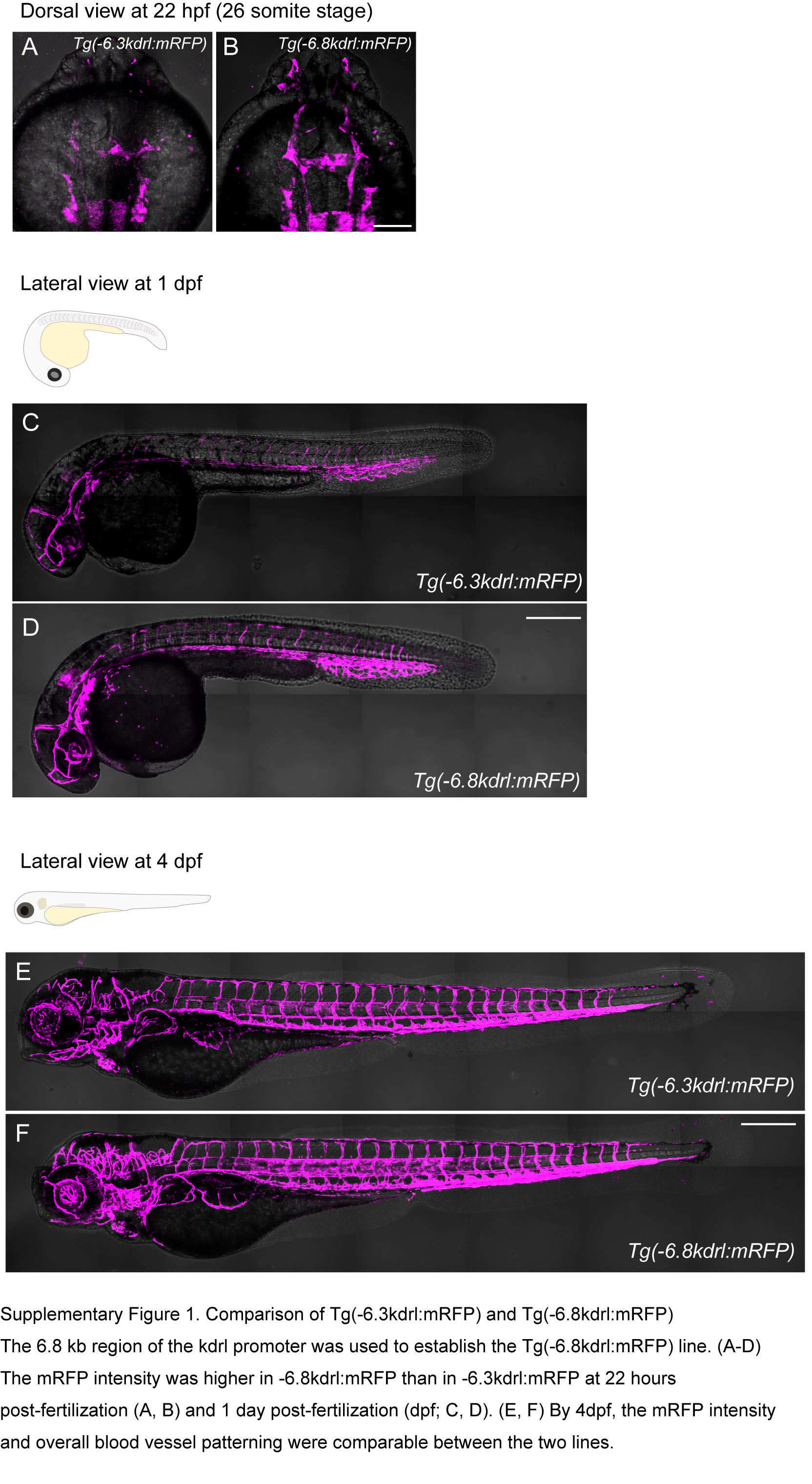

Supplement: kfaf083_Supplementary_Data [file kfaf083_supplementary_data.zip › kfaf083_Supplementary_Data/toxsci-25-0013-File011.tif]

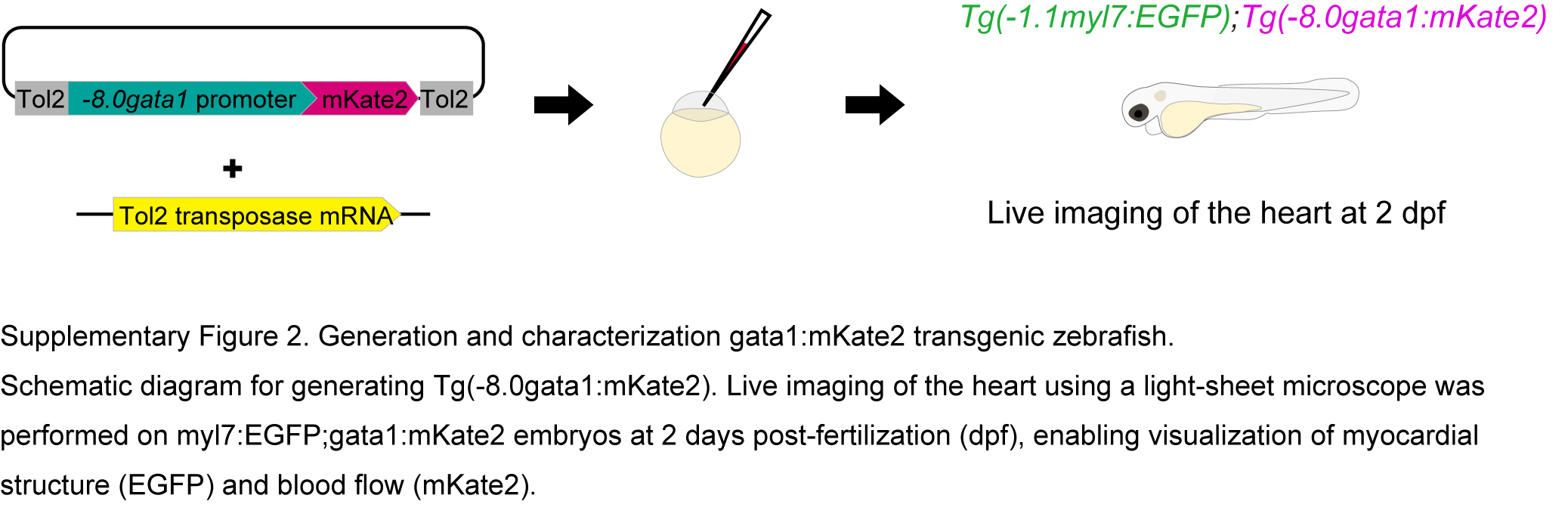

Supplement: kfaf083_Supplementary_Data [file kfaf083_supplementary_data.zip › kfaf083_Supplementary_Data/toxsci-25-0013-File012.tif]
